# Distribution of *Theileria orientalis* in Virginia Market Cattle, 2018–2020

**DOI:** 10.3390/pathogens11111353

**Published:** 2022-11-15

**Authors:** Alex Telionis, Kevin Lahmers, Michelle Todd, Amanda Carbonello, Charles C. Broaddus, Carolynn J. Bissett, Laura L. Hungerford

**Affiliations:** 1 Department of Population Health Sciences, Virginia Maryland College of Veterinary Medicine, Virginia Tech, Blacksburg, VA 24060, USA; 2 Department of Biomedical Sciences and Pathobiology, Virginia Maryland College of Veterinary Medicine, Virginia Tech, Blacksburg, VA 24060, USA; 3 Virginia Tech Animal Laboratory Services (ViTALS), Virginia Maryland College of Veterinary Medicine, Virginia Tech, Blacksburg, VA 24060, USA; 4 Virginia Department of Agriculture and Consumer Services, Richmond, VA 23219, USA

**Keywords:** *Theileria orientalis*, Ikeda genotype, Chitose genotype, prevalence, spatial distribution

## Abstract

*Theileria orientalis*, genotype Ikeda, was recently detected in North America. Determining the emerging distribution of this pathogen is critical for understanding spread and developing management strategies. Whole blood samples were collected from cattle at Virginia livestock markets from September 2018 through December 2020. Animals were tested for *T. orientalis* using a universal and then genotype specific real-time PCR based on the MPSP gene. Prevalence for each genotype was analyzed for temporal trends and mapped by county. Spatial patterns were compared between genotypes and assessed for associations with habitat features, cattle movements through cattle markets and county proximity. Overall, 212 of 1980 samples tested positive for *T. orientalis* with an overall prevalence of 8.7% (172/1980) for genotype Ikeda, 1.8% (36/1980) for genotype Chitose, 0.2% (3/1980) for genotype Buffeli. The Ikeda genotype increased over time in northern and southwestern Virginia markets. The Ikeda and Chitose genotypes occurred in different regions, with little overlap, but for each genotype, spatial distribution was associated with a combination of cattle movements and environmental factors. Genotype specific qPCR testing and surveillance of cattle from across a wide area of Virginia are providing information on temporal, spatial, and other patterns for this emerging disease.

## 1. Introduction

*Theileria orientalis* is a parasitic, unicellular alveolate originally confined to East Asia and Oceania [1,2], but increasingly diagnosed as a cause of bovine infectious anemia in the Mid-Atlantic region of North America [3]. The disease is an economic concern in endemic regions, causing anemia, abortion, and failure to thrive in cattle [2,4] with reduction in the growth rate of beef cattle [5] and substantial reduction in milk volume and quality in dairy cattle [6]. There is currently no approved treatment for *T. orientalis* infection in the USA. Virulence varies widely between the 11 distinct genotypes [7,8] causing asymptomatic to severe disease; the Ikeda genotype is the most clinically concerning [4]. The distinct Chitose and Buffeli genotypes [9], are often found alongside Ikeda variants, though they pose lesser risk to the cattle.

Although the Buffeli genotype has been detected in numerous eastern states since the turn of the century [10,11,12], the more pathogenic Ikeda and Chitose genotypes had not been identified in North America prior to 2017 [3,4] although all three genotypes co-occur in Australasia [13]. The origin of the US introduction remains a mystery, though some have suggested it coincided with the arrival of the Asian Longhorned Tick, *Haemaphysalis longicornis* [14,15,16], which serves as the primary vector for *T. orientalis* in the Eastern Hemisphere [1,17,18]. Since the first confirmed identification in North America in the early 2010s, *H. longicornis* has been found across the East Coast from Connecticut to North Carolina, with widespread distribution in western Virginia and neighboring states [19]. Given the agricultural impact of theileriosis, it is critical to understand the factors which underlie the emerging distribution and spread of this pathogen in North America. Using a recently developed qPCR assay and samples from market cattle collected across the Commonwealth of Virginia, we characterized the prevalence, demographic, and distributional features of *T. orientalis* in cattle in Virginia.

## 2. Materials and Methods

### 2.1. Sampling and Blood Collection

Whole blood samples were collected by Virginia Department of Agriculture and Consumer Services (VDACS) staff beginning in August 2018 with five sites (three Northern, one Central, one Southwest) and scaled up, in January 2019, with seven additional sites (one Northern, three Central, three Southwest). These included 11 of the 12 regularly scheduled, weekly Virginia Livestock Auction sites as well as one additional weekly auction. To gain wide geographic coverage with continuity over time, approximately five adult animals from different lots were sampled during each visit. Fewer samples were collected if there were less than five lots of cattle presented and when staff travel or auction sales were interrupted, mainly due to pandemic COVID restrictions. Whole blood was collected in purple-top BD Vacutainer^®^ blood collection tubes containing EDTA anticoagulant (Becton, Dickinson and Company, Franklin Lakes, NJ, USA). Sex, breed, and radio frequency identification (RFID) tag were recorded. Breeds were also coded as groups (beef, dairy or unknown). Origin of each animal was approximated based on address of the owner, abstracted from market sales records, and geocoded using the Google Maps geocoder API (Available online: https://developers.google.com/maps/documentation/geocoding/overview, accessed on 5 August 2021).

### 2.2. Molecular Detection and Genotyping of T. orientalis

Animals were classified as infected with *T. orientalis* using duplex, real-time PCR, which simultaneously tested for *Anaplasma marginale*, performed as previously published [20]. Briefly, DNA was extracted using the Qiagen DNeasy^®^ Blood and Tissue kit (Qiagen LLC, Germantown, MD, USA). Primers and probes targeted *T. orientalis* MPSP, *A. marginale* msp1b, and a VetMAX™ Xeno™ internal positive control (ThermoFisher Scientific, Waltham, MA, USA), and probes were labeled with NED, FAM and VIC, respectively. Samples were run on an Applied Biosystems™ 7500 Fast Real-Time PCR System (ThermoFisher Scientific) for 45 cycles with an annealing and extension temperature of 60 °C. Samples that were positive for the universal *T. orientalis* qPCR (quantification cycles of less than 45) were then genotyped using a previously published protocol and the same equipment and parameters as above [20,21]. Samples that were positive only after more than 45 amplification cycles were considered equivocal, potentially representing a related gene or an extremely low parasitemia. Samples that were positive using the universal qPCR but negative for all three genotypes were tested with conventional PCR and Sanger sequencing to attempt to characterize their 18s ribosomal RNA subunit and MPSP.

### 2.3. Statistical Analyses

Prevalence estimates, with 95% confidence intervals (CIs), were calculated for each *T. orientalis* genotype for cattle demographic groups. Prevalence Ratios (PR), with 95% exact, unconditional CIs [22], were determined for male versus female, beef versus dairy, breed relative to Angus (the most common breed in our sample) and grouped known breeds (Angus, Hereford, etc.) versus grouped crossbreds. Analyses were completed with R version 4.1.0 [23] using the Exact2x2 package version 1.6.6 [24]. Prevalence for each genotype and the total number of samples collected, by month, were plotted in time for each of the three Virginia cattle market regions [25], because there were too few samples to assess each market individually. Prevalence over time within each market region was assessed for linear trend and then for inflections in slope using adaptive logistic regression splines [26,27] with the Earth 5.3.1 package in R [28].

### 2.4. Spatial Analyses

The number of cattle tested varied between counties; some counties had zero or very few animals tested. Therefore, counties were classified and mapped using ArcMap 10.7 (Esri, Inc., Redlands, CA, USA) for each genotype as having at least one positive sample; having only negative samples; or as unsampled. Spatial codistribution between Ikeda and Chitose genotypes was tested with Tjostheim’s Coefficient [29] after removing counties where neither genotype was found so as to limit artificial similarity due to shared absences [30]. This was done using the SpatialPack package [31] in R.

A network defining connectivity between counties, based on cattle movement through markets, was estimated by first calculating the proportion of geocoded cattle that originated from each county for each market. Since buyer information was not available, bidirectional proportionality of county origin and destination was assumed. Flow was estimated as the product of proportions for each pair of counties divided by the total of these proportions to remove circular movement back to county of origin. Total annual cattle sales per market [32] were then used to scale for market cattle volume differences. Weighted cattle numbers were summed for each county pair across all markets and divided by total market cattle to give the relative weight between each pair of counties. This network was graphed as a force-directed layout to optimize visualization of community structure [33] using Gephi 0.9.2 [34]. The optimized number of communities (clusters) within the network was determined using the Girvan–Newman algorithm [35]. More localized cattle movement was defined by a dichotomous matrix in which counties were considered connected if they shared a border and disconnected if they did not.

Similarity in relevant habitat between pairs of counties was assessed through ecological niche models separately created for the Ikeda and Chitose genotypes using generalized additive models [36] in the ENMTools package in R [37]. Individual geocoded herds with at least one positive animal were used as *Theileria* genotype specific presence points for selecting predictive geospatial variables, while herds with exclusively negative tests were considered to be absence points. Potential predictors included elevation at 30-arc second resolution and 19 remotely sensed bioclimatic variables [38], as well as three new habitat variables (forest, field, and edge densities). The 2019 National Land Cover Dataset [39] was reclassified into forest (NLCD codes 41, 42 and 43) and fields (NLCD codes 71, 81 and 82). Edge-habitat was present where any forest and field blocks intersected [40]. Forest or field area or total linear edge habitat was summed for a 42.45 arc-second diameter circle from the centroid of each bioclimatic cell to cover the entirety of the 30 × 30 arc-second raster cells. Each bioclimatic and habitat variable was resampled and registered to match the resolution of the elevation data using ESRI ArcMap 10.7. Overall similarity was compared between the two genotype niche models using Warren’s I [41], again using the ENMTools package in R. Composite ecological suitability scores for each county and differences between these scores for county-to-county pairs were determined for each genotype.

Join count statistics for dichotomous spatial data [42] were used to assess clustering between counties classified as positive for *T. orientalis*, by genotype, using the SpDep Package for R [43]. The join count test compares the observed number of joins between similar areas to the expected based on total number of areas and possible joins and is more appropriate than continuous autoregressive measures, such as Moran’s I, for binary data [44]. Significance of county connectivity was tested using a series of spatial weight matrices that varied the contribution of cattle movements through markets, local cattle movements, and similarity in genotype ecological niche. The percentage distribution of each was varied from 0 to 100%, always requiring the three to total to 100%, to determine where the combined influence correlated most strongly with the county-level distribution for the *Theileria* genotype [45]. Results were plotted as smoothed spline ternary surface using ESRI ArcMap 10.7.

## 3. Results

**Figure 1:**
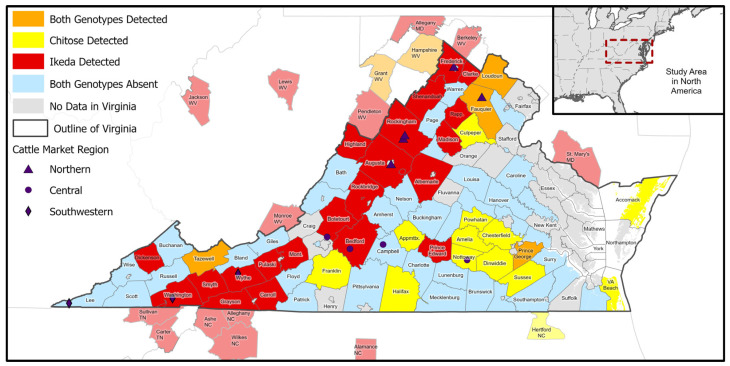
*T. orientalis* genotypes detected across Virginia and neighbors. Counties are colored as red if Ikeda was detected; yellow if Chitose was detected; orange if both genotypes were detected; blue if neither genotype was detected; grey for Virginian counties with no samples. Positive counties outside of Virginia are lighter shades of the same color scheme. Cattle markets are shown as triangles (northern region), circles (central region), and diamonds (southwestern region).

A total of 1993 blood samples were collected from cattle at Virginia markets from August 2018 through December 2020. Thirteen animals were excluded from further analyses because qPCR results were equivocal; positive amplification for *T. orientalis* occurred, but only above the established qPCR cut-off. Of the remaining 1980 samples, 765 were from southwest markets, 744 from northern markets, and 471 from central markets. These were collected on 399 different sampling dates (19 samples were missing collection dates), with an average of 37 sampling dates and 165 samples per market. Samples were collected from southwest markets on 161 different visits, from northern markets on 173 visits, and from central markets on 109 visits. Cattle sampled in Virginia markets came from 82 of the 133 counties/county-equivalents in the Commonwealth and 80 counties in adjacent states (Figure 1). Using qPCR, *T. orientalis* was detected in 212 (10.7%) samples, from 11 of the 12 sale barns surveyed. The Ikeda genotype was the most common, representing 172 (81.1%) of the genotyped samples, present in 8.7% (95% CI = 7.5%, 10.0%) of the 1980 classified samples. The Chitose genotype was found in another 36 cattle, representing 17.0% of the genotyped samples and 1.8% (95% CI = 1.3%, 2.5%) of the classified samples, while the Buffeli genotype was detected only three times, representing 1.4% of the genotyped samples and 0.2% (95% CI = 0.02%, 0.5%) of the classified samples. The genotype of one *T. orientalis* positive sample was not identifiable with the 3 genotype probes nor with conventional PCR for the 18s ribosomal RNA subunit or MPSP. No animals in the study group were found to be infected with multiple genotypes. Among samples with unequivocal test results, 1889 (95.4%) were able to be geocoded, representing 1318 unique herd locations. Ikeda was detected in 132 of these herds (10.0%; 95% CI = 8.5%, 11.8%) and Chitose in 27 herds (1.9%; 95% CI = 1.4%, 3.0%). One unique herd location (<0.1%) had both Chitose and Ikeda positive animals present at the same site. The three Buffeli samples were all collected at the same market on the same date, but the herd locations remained undetermined.

**Figure 2:**
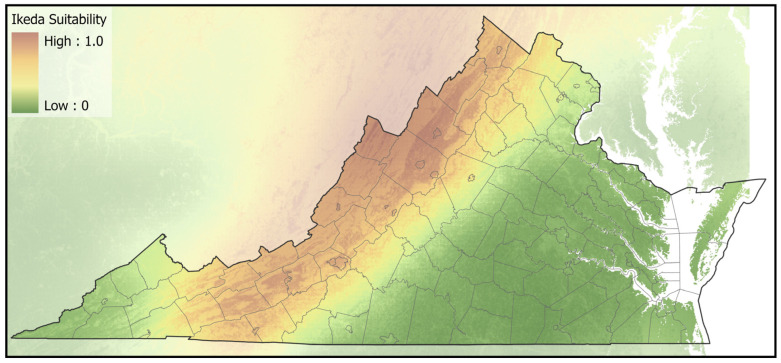
The predicted suitability map for the Ikeda genotype of *T. orientalis*. Orange and brown areas represent optimal habitat, while green areas are predicted to be less suitable habitat.

**Figure 3:**
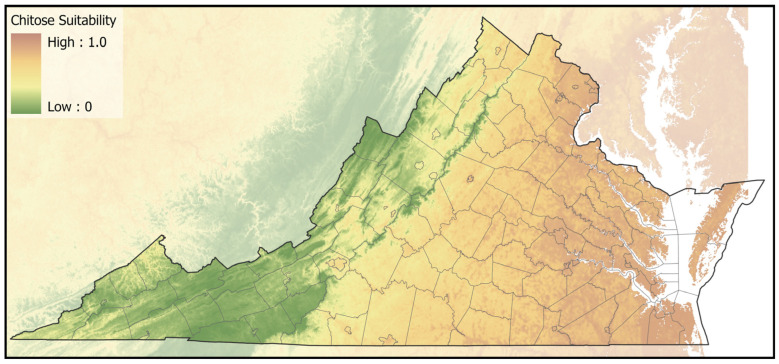
The predicted suitability map for the Chitose genotype of *T. orientalis*. Orange and brown areas represent optimal habitat, while green areas are predicted to be less suitable habitat.

Market cattle that tested positive for *T. orientalis* originated from counties across the Commonwealth and several bordering states (Figure 1). Among 169 represented counties, Ikeda positive herds were found in 52 (30.8%), Chitose positive herds in 20 (11.8%), with six (3.6%) counties having a mixture of herds positive for Ikeda or Chitose. Counties with cattle that tested positive for Ikeda were primarily found in the Appalachian region, running from northeast to southwest along the Interstate 81 corridor on the western side of Virginia. The ecological niche for the Ikeda genotype, developed from locations of individual positive and negative herds, was predominantly in areas with lower forest density that were located in regions with cooler/drier summers (Figure 2, Table S1, Figure S1). Counties with cattle that tested positive for Chitose were primarily concentrated in southcentral and southeast Virginia. The ecological niche for the Chitose genotype, based on locations of individual positive and negative herds, covered lower elevation areas of low forest-herbaceous edge-density, mainly contiguous agricultural fields (Figure 3, Table S1, Figure S2). The county-level distribution of the Ikeda and Chitose genotypes showed significantly less overlap than expected by chance (*p* < 0.001) and the ecological niche patterns were dissimilar (*p* < 0.05).

**Figure 4:**
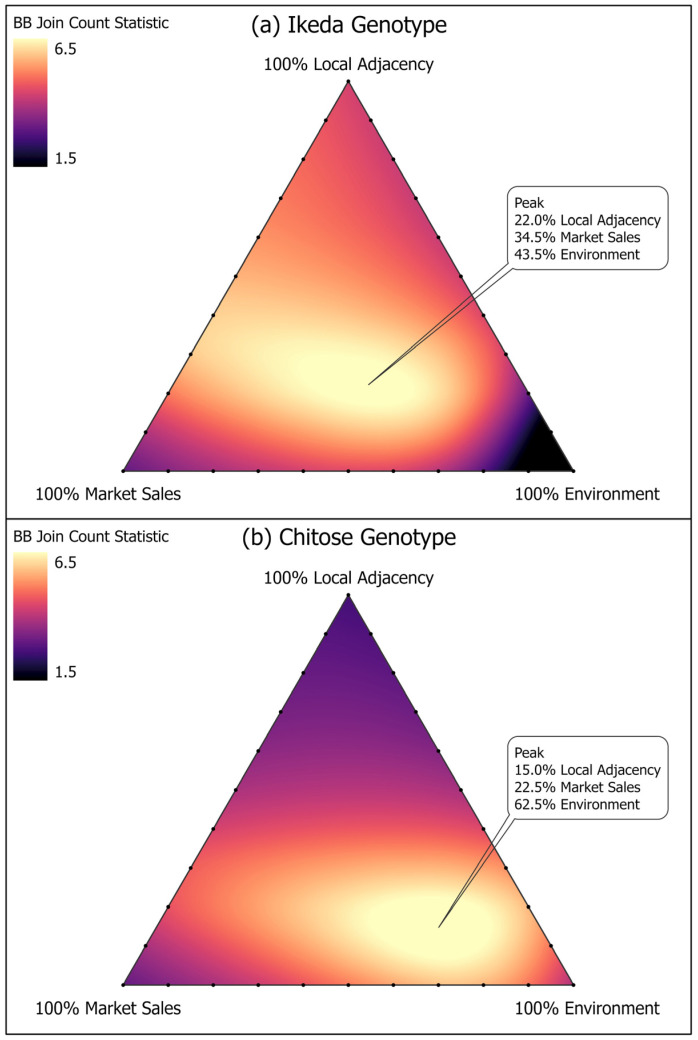
Ternary graphs showing the weighted contribution of market sales, adjacency, and environmental similarity to county-level clustering of Ikeda (**a**) and Chitose genotypes (**b**). The color at each point represents the join count statistic, measuring the spatial autocorrelation for the combination of factors. The brightest peaks represent the optimal contribution that best explained the observed spatial association for each genotype.

Counties were extensively linked through shared cattle markets (Figure S3), with each county connected to a median of 40 other counties with a range of 7 to 113 links per county. Weights between pairs of counties ranged from 0 to 832, with a median of 0, since only 28% of county-pairs were connected through cattle markets. Counties with at least one Ikeda positive herd had significantly greater connectivity with other positive counties through cattle markets and also were more likely to be adjacent to other positive counties. This relationship became stronger when the counties had similar proportions of forested area and the cool/dry summers characterized by the ecological niche profile (Figure 4a). The strongest association was with a contribution of 34.5% for market movements, 22% for adjacency, and 43.5% for niche similarity. Counties with at least one Chitose positive herd had similar proportions of the lower elevation, agricultural field ecological niche, with connectivity through cattle markets and local flows also statistically significant (Figure 4b). The strongest relationship was with a contribution of 62.5% for niche similarity, 22.5% for market movements, and 15% for local movements.

The number of animals that tested positive for *T. orientalis* genotypes was small, limiting our power to assess demographic differences. Although beef cattle made up the majority of animals testing positive for Ikeda, prevalence was higher among dairy cows, approaching significance (*p* = 0.06; Table 1). Among individual breeds, prevalence was significantly higher in cross-bred cattle than among Angus (*p* < 0.01), with increased prevalence in Holstein cattle approaching significance (*p* = 0.09). With only 36 positive Chitose results, we lacked power to detect demographic differences, however the direction of the prevalence relationships tended to be opposite of those seen with Ikeda.

**Figure 5:**
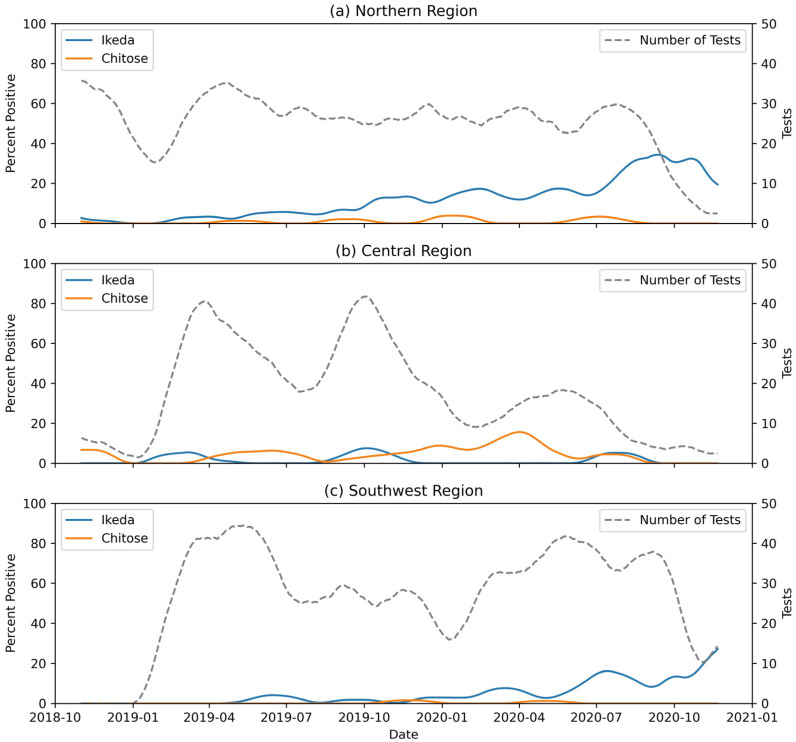
Three month moving average of sample positivity by genotype for the northern (**a**), central (**b**) and southwestern (**c**) market regions. The total volume of testing is shown with grey dashed line on each graph.

In fall 2018, at the start of the study, the Ikeda genotype was detected in northern markets and the Chitose genotype in northern and central markets. Ikeda and Chitose were detected in fall 2019 in the southwestern markets after sampling began there in January 2019. Temporal prevalence patterns differed between Ikeda and Chitose and between market regions of Virginia. In the northern markets, the prevalence of the Ikeda genotype increased each month (Figure 5a) by about 14% of the previous month (OR = 1.14; 95% CI = 1.10, 1.19). In the southwestern markets (Figure 5b), Ikeda prevalence also increased about 14% each month from August 2018 through April 2020 (OR = 1.14; 95% CI = 1.02, 1.26), followed by a sharper increase of about 38% compared to the previous month from April 2020 through December 2020 (OR = 1.38; 95% CI = 1.32, 1.83). For the Chitose genotype, there were only sporadic positives in the northern and southwestern regions with no consistent trends. For the central group of markets, there were no significant trends for either genotype (Figure 5c).

## 4. Discussion

We investigated the occurrence and distribution of *T. orientalis*, an emerging cause of bovine infectious anemia, in samples from cattle marketed across Virginia. We found the Ikeda, Chitose, and Buffeli genotypes in Virginia and neighboring states with Ikeda in 8.7% of our samples and Chitose in just under 2%. No cattle were co-infected with more than one genotype in our study. The prevalence of the Ikeda genotype increased over time in northern and southwestern Virginia cattle markets. The Ikeda and Chitose genotypes occurred in different regions, with little overlap, and with disparate environmental predictors. Characteristics of infected cattle also varied for the two genotypes. Despite these differences, connectivity based on cattle movements, through markets and locally, and on environmental factors contributed to the distribution of *T. orientalis*, for both genotypes.

*T. orientalis* Ikeda was first recognized in the USA as a clinical outbreak in a beef herd in west-central Virginia in fall 2017 [3]. Our detection of both the Ikeda and Chitose genotypes from multiple cattle across Virginia in fall 2018 and the temporal patterns in market groups show that both genotypes are established, and that Ikeda is spreading. This increasing presence of a new pathogen may not have been recognized in other herds, as asymptomatic infection is common [4] and anaplasmosis, which has similar clinical signs, is also present in Virginia [46]. In New Zealand, *T. orientalis* Ikeda was first detected in spring 2012 and spread rapidly across much of the northern island within two years [47]. In Australia, the Ikeda genotype was initially detected in 2006 and spread rapidly across most states [48].

The Ikeda genotype is found in highest cattle density areas in Virginia, composed mainly of cow-calf operations but also including those counties with the highest numbers of dairy cows [49]. Scotch-Irish and German settlers in this region historically purchased and fattened cattle, providing beef for northern markets through cattle drives to Baltimore and Philadelphia [50]. The steep, lush pastures and limited workforce led to similar management practices across this region of Appalachia [51]. This continuing practice of buying and assembling cattle to feed and resell later can amplify disease spread. The strong association of Ikeda with cattle movements, through cattle markets or county adjacency, matches dissemination in New Zealand, where both long distance and local cattle translocations were important [47]. One advantage of testing cattle moving through the market system, as in our study, is that these represent animals with potential to enter a new herd. In this region, bulls are generally also moved into cow herds for the breeding season, providing another possible route of spread through cattle movement.

Although counties with Ikeda positive cattle were significantly linked through cattle movements, these associations were strongest for counties sharing similarity in the forest dense, cooler/drier summer niche. The importance of both movements and environmental features has been shown for *T. orientalis* in New Zealand and Australia [2,47] as well as for other vector-borne diseases of cattle [44,52]. The direct effect of climate should be limited on the parasite itself, which can spend the entirety of its lifecycle within a host and, potentially, within a vector. While the ecological niche could reflect locations favorable for cattle rearing, we contrasted locations of Ikeda positive and negative herds to control for overall effects of cattle distribution in environmental factor selection. These niches could reflect locational suitability for other important hosts and/or vectors within the pasture-based management in this region. *H. longicornis* has been collected from Virginia animals with theileriosis [20] and is a demonstrated vector of the Ikeda genotype, both internationally and using Virginian strains of both tick and parasite [4]. Before the start of our survey in 2018, the tick had already been passively reported from 20 counties along Virginia’s northernmost to southwestern border and is currently found in 38 counties [19], mainly in the same western region as the Ikeda positive animals described in this study. In New Zealand, the most rapid dissemination of the Ikeda genotype was in areas where tick vectors were already established. Recently, Cumbie et al. [53], in a longitudinal study of *H. longicornis* at sites in central Appalachian Virginia, found forested habitats, temperature, and precipitation important predictors of tick abundance, aligning with our associations for the presence of Ikeda infections in cattle. Since pasture grazing still predominates in this region, even for certain production intervals for dairy cattle, opportunities for exposure to vectors and other cattle are abundant.

Though Chitose commonly co-occurs with Ikeda in other countries [54], we found no co-infected animals in our study. The Chitose genotype was first identified in Virginia through our study, so its historical distribution in the US is unknown. In New Zealand, Chitose was recognized as causing mild disease in 1982 with Ikeda likely introduced decades later, but in the same regions [47]. In Australia, one of the two genetic clusters of Chitose (B) was reported more often alone, while in Queensland, a second cluster (A) was more commonly found in cases of co-infection after the introduction of Ikeda [55]. The patterns in the US may also represent separate introductions of Chitose and Ikeda at disparate locations and times.

Chitose genotype clustered among counties that were significantly connected with each other through cattle movements but separate from the Ikeda counties. Cattle in southcentral Virginia were introduced by English settlers from the coast [50] and remain at a lower density, separated by mountain ridges and limited transportation routes from the Ikeda-rich Appalachian region [49]. This may have slowed dissemination of this genotype and may also account for the trend toward different breed associations than those found for Ikeda.

Overall, the Chitose genotype was less prevalent than Ikeda within Virginia market cattle. *H. longicornis* has been shown to have lower vector competence for Chitose than for Ikeda in mixed infections [55], which could lead to lower prevalence. Alternatively, since the Chitose distribution and the ecological niche factors, specifically lower elevation and more contiguous agricultural fields, did not overlap with those reported for *H. longicornis*, this may indicate a different vector or different means of transmission for Chitose. Other *Theileria* species are spread by *Amblyomma*, *Dermacentor*, and *Rhipicephalus* species, including those found in Virginia: *A. americanum*, *D. variabilis*, and *R. sanguineous* [56]. Although only studied with Ikeda, transmission directly using blood was slower and less effective than tick transmission [4], which could also account for lower prevalence in areas with fewer tick vectors.

Although this study provides important, initial information about the distribution of *T. orientalis* genotypes in this region, the limited number of cattle sampled and the selection of one animal from only up to five lots at a sale makes it likely that the breadth of occurrence was underestimated. However, most studies of emerging animal diseases are based on smaller samples of clinical cases or within a few herds, so this large, geographically broad, surveillance study capturing more than 1300 herds shows the importance and wide distribution of this recently recognized infection. Virginia dairy cattle were also under-represented at market sales but those sampled tended toward having a higher prevalence, so this production system should have further study. If infection was associated with loss of condition, abortion, or calf loss, prevalence could be higher in culled market cows than those remaining in herds. Additionally, detection of *Theileria* in healthy US market cattle could represent animals with recent infections, with mild disease, or recovered carriers, making the temporal pattern for onset of individual, herd, or regional spread unknown. Study of clinical cases and outbreaks of theileriosis and surveys of veterinarians and producers about knowledge, occurrence, and management practices across affected and unaffected regions of Virginia are needed to better understand and manage this disease.

This study provides an initial description of occurrence patterns of *T. orientalis* genotypes Ikeda and Chitose in Virginia and neighboring states. Surveillance for an introduced livestock disease is challenging because the prevalence and determinants of distribution may differ from those in other parts of the world. These genotypes, especially Ikeda, have significant negative economic and welfare effects on cattle internationally and this information will help with development of management strategies for the US. Availability of a duplex test for *A. marginale* and multiplex test for *T. orientalis* genotypes [20] provides an important tool for detecting, differentiating, and managing both diseases

## Figures and Tables

**Table 1:** The number of cattle that tested positive and negative for each *Theileria* genotype, with the prevalence ratios and confidence intervals for selected demographic characteristics.

Characteristic	Ikeda Positive (*n* = 172)	Chitose Positive (*n* = 36)	*Theileria* Negative (*n* = 1768)	Ikeda		Chitose	
				PR ^1^	95% CI ^2^	PR ^1^	95% CI ^2^
Sex							
Female	172	36	1746	Referent ^3^		Referent ^3^	
Male	0	0	7	0.00	(0.00, 4.98)	0.00	(0.00, 20.67)
Unknown	0	0	15	0.00	(0.00, 2.82)	0.00	(0.00, 11.45)
Purpose							
Beef	154	36	1635	Referent ^3^		Referent ^3^	
Dairy	18	0	107	1.67	(0.97, 2.61)	0.00	(0.00, 1.72)
Unknown	0	0	26	0.00	(0.00, 1.83)	0.00	(0.00, 6.62)
Breed							
Angus	95	24	1093	Referent ^3^		Referent ^3^	
Cross-Bred	49	8	337	1.59 *	(**1.12**, **2.19**)	1.08	(0.44, 2.34)
Charolais	4	3	77	0.62	(0.16, 1.59)	1.75	(0.39, 5.32)
Holstein	11	0	69	1.72	(0.84, 3.02)	0.00	(0.00, 2.61)
Hereford	5	1	72	0.81	(0.26, 1.88)	0.64	(0.03, 3.67)
Other	8	0	94	0.98	(0.39, 1.93)	0.00	(0.00, 1.94)
Unknown	0	0	26	0.00	(0.00, 1.91)	0.00	(0.00, 6.56)

^1^ PR = Prevalence Ratio. ^2^ CI = Confidence Interval. ^3^ Referent = Category which served as the base for calculation of Prevalence Ratios for all comparisons within a group of characteristics. Statistically significant results (*p* < 0.05) are shown in bold and noted by an astrix next to the value.

## Data Availability

The data presented in this study are not publicly available due to the sensitive nature of the disease diagnoses. Even though the dataset has limited identifiers, we believe that there remains the possibility of deductive disclosure of herds due to market and county locators and some breed characteristics. Portions of the data can be shared through a data use agreement.

## Supplementary Materials

The following supporting information can be downloaded at: https://www.mdpi.com/article/10.3390/pathogens11111353/s1, Table S1: Response curves for the environmental factors relating the presence and absence of the Ikeda genotype in the generalized additive model; Figure S1: Response curves for the final variables in the generalized additive model relating the presence and absence of the Ikeda genotype to environmental factors; Figure S2: Response curves for the environmental factors relating the presence and absence of the Chitose genotype in the generalized additive model; Figure S3: Force-directed network showing Girman-Newman clusters of counties connected by cattle sales in Virginia
