# Supplementary material for: Distribution of Theileria orientalis in Virginia Market Cattle, 2018–2020
Source: Pathogens. 2022 Nov 15;11(11):1353. doi: 10.3390/pathogens11111353 (PMC9695988; doi:10.3390/pathogens11111353)
Supplement: Supplementary file 1 [file pathogens-11-01353-s001.zip › pathogens-1936026-supplementary.pdf]

Distribution of *Theileria orientalis* in Virginia market cattle, 2018-2020
Supplementary Material

Table S1: Variable importance for the final generalized additive model relating the presence and absence of the Ikeda genotype to environmental factors. These were calculated using the enmtools.vip function [37] using the "permute" method with 100 simulations each. Values were scaled to 100%.

Variable	Importance for Ikeda	Ikeda Rank	Importance For Chitose	Chitose Rank
BIO1 (Annual Mean Temperature)	0	-	0	-
BIO2 (Mean Diurnal Range)	0	-	0	-
BIO3 (Isothermality)	0	-	0	-
BIO4 (Temperature Seasonality)	0	-	0	-
BIO5 (Max Temperature of Warmest Month)	**0.1102**	**2**	0	-
BIO6 (Min Temperature of Coldest Month)	0	-	0	-
BIO7 (Temperature Annual Range)	0	-	0	-
BIO8 (Mean Temperature of Wettest Quarter)	0	-	0	-
BIO9 (Mean Temperature of Driest Quarter)	0	-	0	-
BIO10 (Mean Temperature of Warmest Quarter)	0	-	0	-
BIO11 (Mean Temperature of Coldest Quarter)	0	-	0	-
BIO12 (Annual Precipitation)	0	-	0	-
BIO13 (Precipitation of Wettest Month)	0	-	0	-
BIO14 (Precipitation of Driest Month)	0	-	0	-
BIO15 (Precipitation Seasonality)	0	-	0	-
BIO16 (Precipitation of Wettest Quarter)	0	-	0	-
BIO17 (Precipitation of Driest Quarter)	0	-	0	-
BIO18 (Precipitation of Warmest Quarter)	**0.8276**	**1**	0	-
BIO19 (Precipitation of Coldest Quarter)	0	-	0	-
Elevation	0	-	**0.9503**	**1**
Forest-Herbaceous Edge Density	0	-	**0.0497**	**2**
Field Density	0	-	0	-
Forest Density	**0.0622**	**3**	0	-

Distribution of *Theileria orientalis* in Virginia market cattle, 2018-2020
Supplementary Material

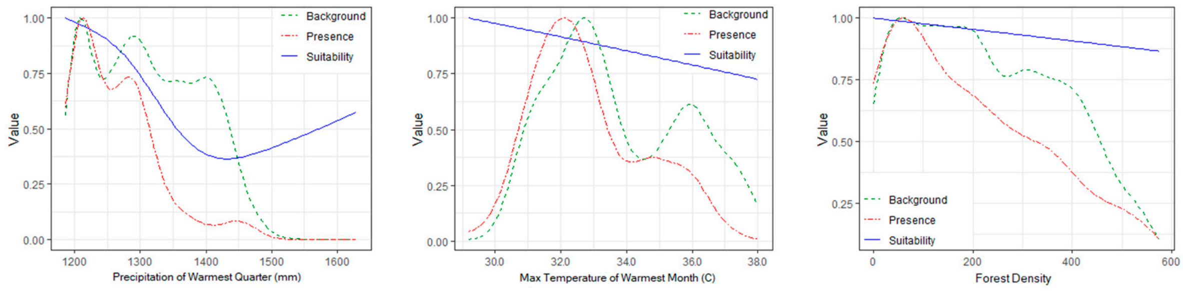

(a) (b) (c)

Figure S1: Relevant response curves for the final generalized additive model relating the presence and absence of the Ikeda genotype to environmental factors. Precipitation of the warmest quarter (a), maximum temperature of the warmest month (b), and forest density (c) were the only primary contributors to the model.

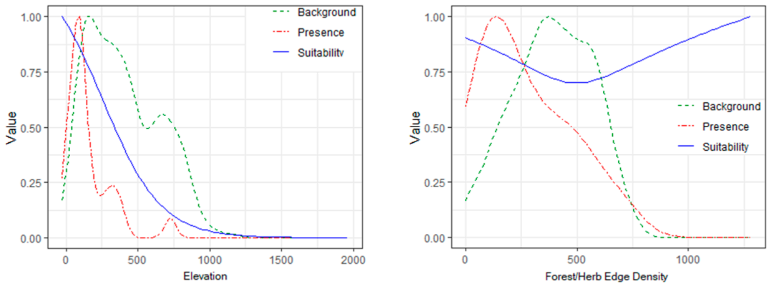

(a) (b)

Figure S2: Response curves for the final generalized additive model relating the presence and absence of the Chitose genotype to environmental factors. Elevation (a) and forest-herbaceous edge-density (b), were the primary contributors to the model.

Distribution of *Theileria orientalis* in Virginia market cattle, 2018-2020
Supplementary Material

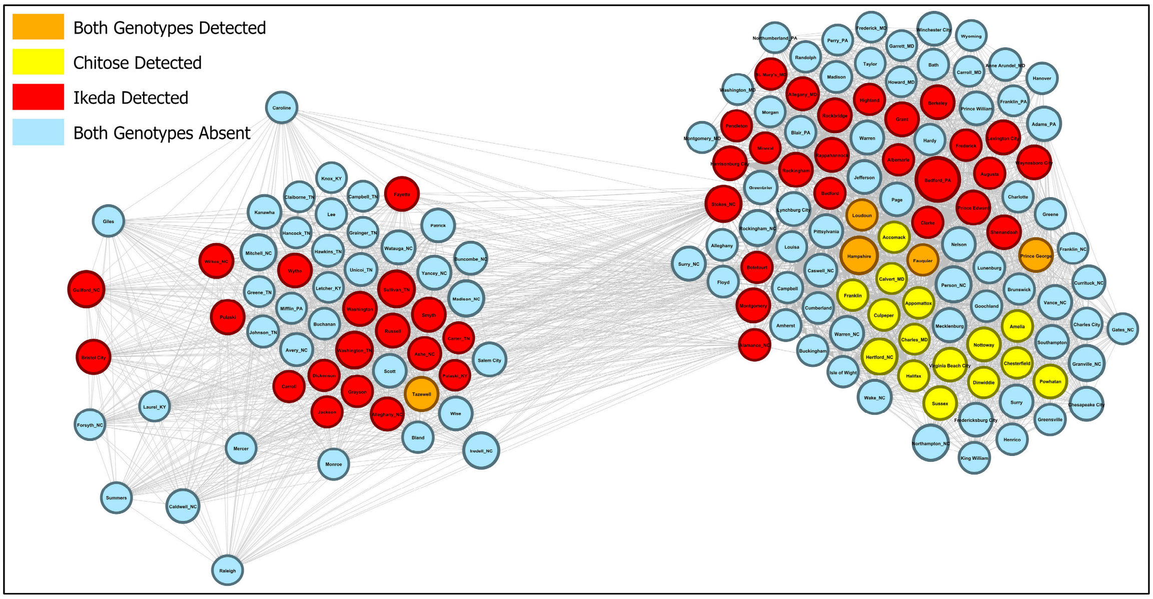

Figure S3: Force-directed diagram of cattle market connections between pairs of counties created using Gephi 0.9.2 [34]. The force-directed diagram method pulls nodes closer together if they are connected, and even more by greater weighted connections, and pushes them apart if they are not connected, aiding visualization of the market connectivity between pairs of nodes [33]. Nodes represent counties colored by the presence or absence of genotypes; red for Ikeda, yellow for Chitose, orange for both, blue for neither. Edges represent the magnitude of cattle sales between pairs of counties, with closer nodes sharing more cattle sales.
